# “From Waste to Wonder”: Comparative Evaluation of Chinese Cabbage Waste and Banana Peel Derived Hydrogels on Soil Water Retention Performance

**DOI:** 10.3390/gels10120833

**Published:** 2024-12-18

**Authors:** Yufan Xie, Yuan Zhong, Jun Wu, Shiwei Fang, Liqun Cai, Minjun Li, Jun Cao, Hejie Zhao, Bo Dong

**Affiliations:** 1 College of Resources and Environment Sciences, Gansu Agricultural University, Lanzhou 730070, China; 18809453467@163.com (Y.X.); 13892801256@163.com (S.F.); cailq@gsau.edu.cn (L.C.); l1174219037m@163.com (M.L.); dongbobby@163.com (B.D.); 2 State Key Laboratory of Aridland Crop Science, Gansu Agricultural University, Lanzhou 730070, China; zhongy@gsau.edu.cn; 3 Research Center for Water-Saving Agriculture in Gansu Province, Lanzhou 730070, China; 4 Agricultural Technical Extension Station of Gannan Tibetan Autonomous Prefecture, Gannan 747000, China; 18109411990@163.com (J.C.); 13321240991@163.com (H.Z.)

**Keywords:** hydrogel, Chinese cabbage waste, banana peel, acrylic acid, acrylamide, water retention, swelling capacity, drought stress

## Abstract

Under the increasing severity of drought issues and the urgent need for the resourceful utilization of agricultural waste, this study aimed to compare the soil water retention properties of hydrogels prepared from Chinese cabbage waste (CW) and banana peel (BP) using grafting techniques with acrylic acid (AA) and acrylamide (AAm). Free radical polymerization was initiated with ammonium persulfate (APS), and N, N′-methylene bisacrylamide (MBA) served as the crosslinking agent to fabricate the grafted polymer hydrogels. The hydrogels were subjected to detailed evaluations of their water absorption, reusability, and water retention capabilities through indoor experiments. The optimal hydrogel was identified and its applicability in wheat seedling growth was assessed. The findings revealed that the CW-gel, with an equilibrium swelling ratio of 551.8 g/g in ultrapure water, demonstrated remarkable performance and sustained a high water retention of 57.6% even after drying, which was markedly superior to that of the BP-gel. The CW-gel with the best comprehensive properties significantly improved water retention in sandy soil by 78.2% and prolonged the retention time by five days, indicating its potential for long-term irrigation management. In contrast, the BP-gel showed better performance in clay soil, with an increased water-holding capacity of 43.3%. The application of a 1.5% CW-gel concentration under drought stress significantly improved wheat seedling growth, highlighting the role of hydrogels in agriculture and providing a new path for sustainable water resource management in dryland farming.

## 1. Introduction

In the context of global climate change, the increasing frequency and severity of droughts pose a significant challenge to the global agricultural production system [1]. The scarcity of water resources not only limits crop growth and development but also threatens global food security and ecosystem stability at a deeper level [2]. In this context, the research of efficient water resource management and utilization technologies has become the key to ensuring the sustainable development of agriculture. In recent years, the preparation of multifunctional hydrogels from agricultural wastes has attracted considerable attention from both academia and industry as an innovative strategy for resource recycling and water conservation [3,4]. Hydrogels exhibit excellent water absorption properties by forming a three-dimensional hydrophilic network structure through physical or chemical cross-linking mechanisms [5]. These materials are capable of rapidly absorbing large amounts of water without dissolving, and then slowly releasing the water through a controlled release mechanism. Hydrogels can be used as soil conditioners and controlled-release fertilizer carriers in agriculture, significantly improving the water-holding capacity and water-use efficiency of the soil by improving the physical structure of the soil [6]. This technology path can not only effectively reduce the dependence of agriculture on water resources, but also significantly improve the drought-resistant performance of crops, which is of great significance in enhancing the adaptability and resilience of agricultural production and promoting the efficient use of water resources [7]. The application of superabsorbent polymer hydrogels opens a new way for sustainable agricultural development under the current situation of increasing global water stress.

In recent years, the resource use of agricultural waste has become a research hotspot, where the conversion of agricultural waste into hydrogels with high water retention and biodegradability not only helps to improve the soil moisture status and crop growth conditions but also meets the concept of sustainable development. Simeng et al. (2020) [8] have demonstrated that agricultural waste-derived hydrogels contribute to the reduction of irrigation requirements, enhancement of nutrient retention, and increase in crop yield, thereby promoting environmental sustainability and the development of the agricultural circular economy. Furthermore, the economic viability of employing these hydrogels as soil amendments has been analyzed, with an emphasis on their potential to decrease irrigation needs, improve nutrient retention, and enhance crop productivity. Despite the limitations, such as high energy consumption, high cost, and low recovery rate of nanocellulose, this research provides robust evidence for the potential growth of the agricultural hydrogel market. Miljković V et al. (2021) [9] found that carboxymethyl cellulose (CMC) hydrogels with super-absorbent properties can be synthesized through the chemical modification of cellulose derived from plant waste materials. The paper offers insights into the potential of utilizing waste plant resources to produce CMC super-absorbent hydrogels for sustainable agriculture. However, the primary limitation of the study is that it primarily concentrates on the chemical modification process and the biodegradability of the material, and it lacks a comprehensive assessment of the environmental impact and economic viability of the material, which constrains its dissemination and adoption in practical applications. Zhang et al. (2021) [10] successfully prepared a novel superabsorbent polymer gel from white cabbage, which showed excellent water uptake and salt resistance in various solutions. However, this study only focused on a single vegetable waste did not compare other vegetable wastes, and did not systematically investigate the effects of different monomer ratios, initiator, and cross-linking agent contents on the water retention properties. Zgallai et al. (2023) [11] investigated the application of organic waste compost and commercial water retention agents in semi-arid soils and found that both were effective in improving soil water retention and plant growth, especially the organic waste compost, which performed better and improved soil water retention and plant growth. Both were found to be effective in improving soil water retention and plant growth, with organic waste compost performing better and improving soil fertility. However, the limitations of the study are that it was only conducted under specific soil conditions and the results may not be generalizable. This paper addresses these research gaps and further explores the potential of hydrogels produced from agricultural waste and their application in agriculture.

Chinese cabbage is one of the commonest vegetables on the table, yet the outer leaves and non-edible parts produced during its processing are often considered waste. Globally, approximately 1.3 billion tons of food and vegetables are discarded annually, while vegetable production is projected to reach 2.2 billion tons by 2025, according to data from the Food and Agriculture Organization (FAO). In China, approximately 34.2 million tons of Chinese cabbage are discarded annually, the majority of which is deposited directly into landfills without prior treatment [12]. This practice not only results in the waste of valuable resources but also has a detrimental impact on the environment. CW is rich in cellulose and pectin, acts as a natural polymer, and forms a stable three-dimensional network structure, resulting in hydrogel materials with excellent water absorption and water retention [13]. Similarly, BP, another agricultural waste, is also rich in cellulose and pectin, and has similar biological properties to CW, but is unique. The fiber structure and pectin content of BP make it a high-quality raw material for the production of high-performance hydrogels [14]. Its water content and specific fiber arrangement provide additional optimization of the material’s water absorption and retention. The transformation of BP in the treatment and modification process not only reduces environmental pressure but also adds new dimensions to the performance of hydrogel materials through its natural properties, such as enhanced flexibility and biocompatibility, to meet the requirements of applications in different fields [15].

In the study, we synthesized novel CW-acrylic acid-acrylamide highly absorbent gel (CW-(AA-AAm) gel) and BP-acrylic acid-acrylamide highly absorbent gel (BP-(AA-AAm) gel) by grafting copolymerized monomers onto CW and BP, respectively, and chemically cross-linking them. By modifying the ratio of initiator, monomer, and cross-linker, the variations in soil water retention characteristics of hydrogels produced from diverse agricultural waste materials were investigated, thereby offering substantial support for the advancement of agricultural water conservation and soil moisture retention in arid regions.

## 2. Results and Discussion

### 2.1. Synthesis of (AA-AAm) Gels

**Figure 1:**
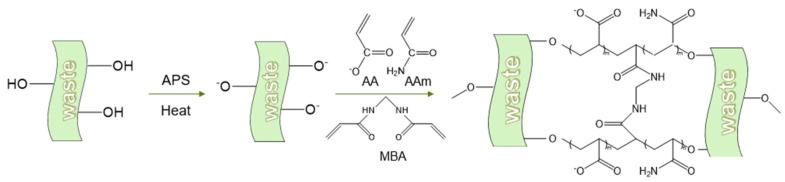
Synthetic Route of Cross-linked (AA-AAm) Copolymers as Water Super-absorbent Hydrogels.

For the synthesis of the (AA-AAm) gels, CW and BP were copolymerized with AA and AAm, respectively, and oxidized with cellulose and pectin by radical polymerization, grafted with ammonium persulphate and then initiated by radical polymerization in the presence of a small amount of MBA as the crosslinking agent. Finally, AA and AAm were randomly grafted onto the agricultural waste suspension, and five CW-(AA-AAm) gels and five BP-(AA-AAm) gels were synthesized (Figure 1).

### 2.2. FTIR Spectra Characterization Analysis

**Figure 2:**
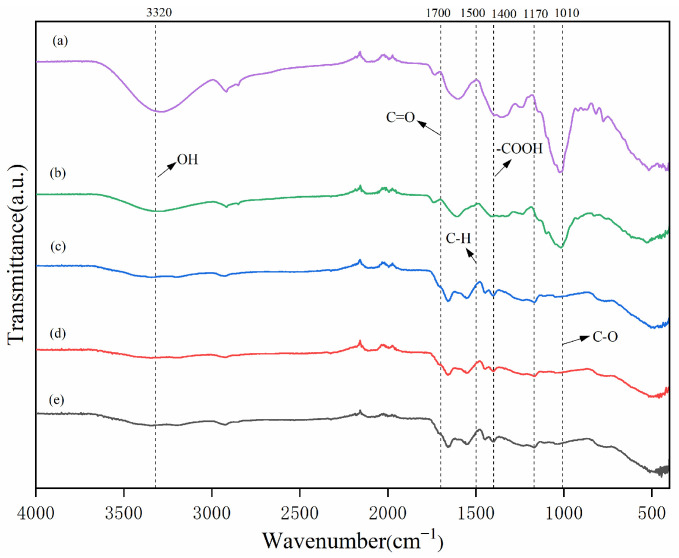
FTIR spectral characterization of (a) CW, (b) BP, (c) CK, (d) BP-(AA-AAm) gel, and (e) CW-(AA-AAm) gel. The term “CK” denotes a hydrogel that has been prepared without incorporating any agricultural waste materials, serving as a control in our experiments to isolate the effects of the added waste components.

A distinctive absorption peak was observed at 3190 cm^−1^, indicating the presence of amide groups in the sample and confirming the corresponding chemical structure (Figure 2). The distinctive absorption peak at 1700 cm^−1^ indicates the C=O functional group undergoing a stretching vibration, thereby confirming the incorporation of acrylic acid (AA) and the occurrence of an esterification reaction [16]. Absorption peaks associated with the C-H bending vibration of the amide group and the asymmetric stretching vibration of the carboxylic acid group were observed at 1500 cm^−1^ and 1400 cm^−1^, respectively. Notably, the amide’s C-H absorption peak exhibited a notable displacement following the reaction, indicating the reconfiguration and reorganization of the amide group during the chemical modification process [17]. The distinctive absorption peaks of asymmetric C-O-C bridges were observed in the CW-(AA-AAm) gel and BP-(AA-AAm) gel at 1170 cm^−1^. Furthermore, the absorption peak observed at 1010 cm^−1^ was attributed to the C-O stretching vibration of the alcohol hydroxyl group. However, the peak was barely discernible after the reaction.

**Figure 3:**
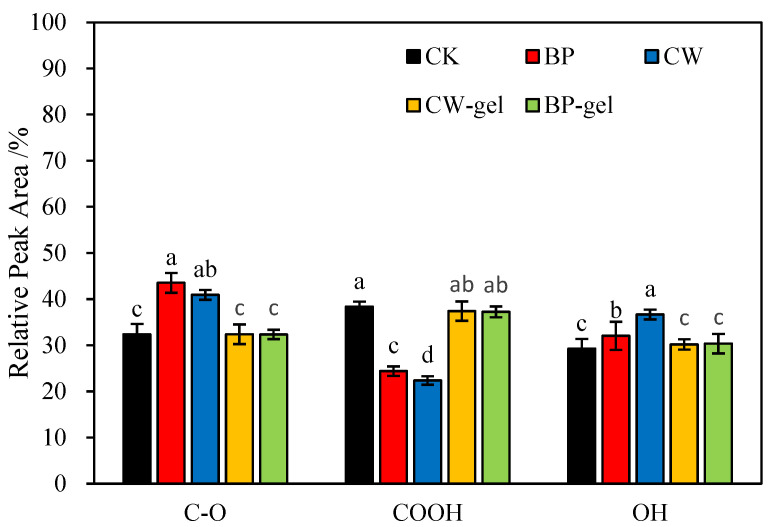
Comparative Analysis of Relative Peak Areas for C-O, COOH, and OH Groups in Waste Materials (CW, BP) and Hydrogels (CK, CW-gel, BP-gel). In the bar graph, letters (a–d) indicate the degree of significant differences among the groups represented by each bar, with “a” denoting the group with the highest level of significance, followed by “b”, “c”, and “d” representing groups with decreasing levels of significance.

As illustrated in Figure 3, a comparison of the relative peak areas of the components in CW, BP, CK, CW-gel, and BP-gel reveals that the C-O content in BP is markedly higher than in the other groups. This is attributed to the inherent chemical composition of BP [18]. Furthermore, the highest content of COOH was observed in CK, which may be attributed to the fact that CK retained a greater number of carboxyl functional groups during the synthesis process. The elevated OH content in CW can be attributed to the high concentration of hydroxyl functional groups present in this sample [19]. The COOH and OH contents in CW-gel and BP-gel exhibited similarity to that of CK, suggesting that the graft polymerization treatment has preserved the functional group contents of the raw materials to a certain extent.

The analytical results of IR spectra demonstrated notable alterations in the molecular structure of the matrix throughout the chemical modification process. The presence of absorption peaks associated with the N-H stretching vibration indicated the successful incorporation of acrylamide, reflecting the stable presence of the amide chain in the modified material. Conversely, the conspicuous C=O stretching vibration peak indicates the effective introduction of acrylic acid, thereby confirming the success of the esterification reaction [20]. The alterations in frequency and intensity of the amide and carboxylic acid groups’ absorption peaks illustrate the increase or decrease of these functional groups throughout the chemical modification process, thereby demonstrating the changes in the molecular structure resulting from the chemical reaction. For instance, the displacement of the C-H absorption peak of the amide suggests a change in its environment during the modification reaction, which may indicate intermolecular interactions, hydrogen bond formation, or other chemical interactions [21]. The appearance of the characteristic absorption peak of the asymmetric C-O-C bridge at 1170 cm^−1^ is an important indication of the success of chemical modification [22]. This suggests that N, N′-methylene bisacrylamide (MBA) effectively participated in the reaction as a cross-linking agent, thereby promoting the formation of the reticular structure. The stabilization of the reticular structure is crucial for enhancing the mechanical properties and chemical stability of the material, particularly in a multitude of applications [23]. This can lead to an improvement in the durability and reliability of the material. The disappearance of the absorption peak at 1010 cm^−1^ unambiguously signifies the transformation of the alcohol hydroxyl group. This suggests that the alcohol hydroxyl group was successfully incorporated into the chemical modification reaction, potentially undergoing conversion into new functional groups or participating in the cross-linking process.

### 2.3. SEM Characterization Analysis

**Figure 4:**
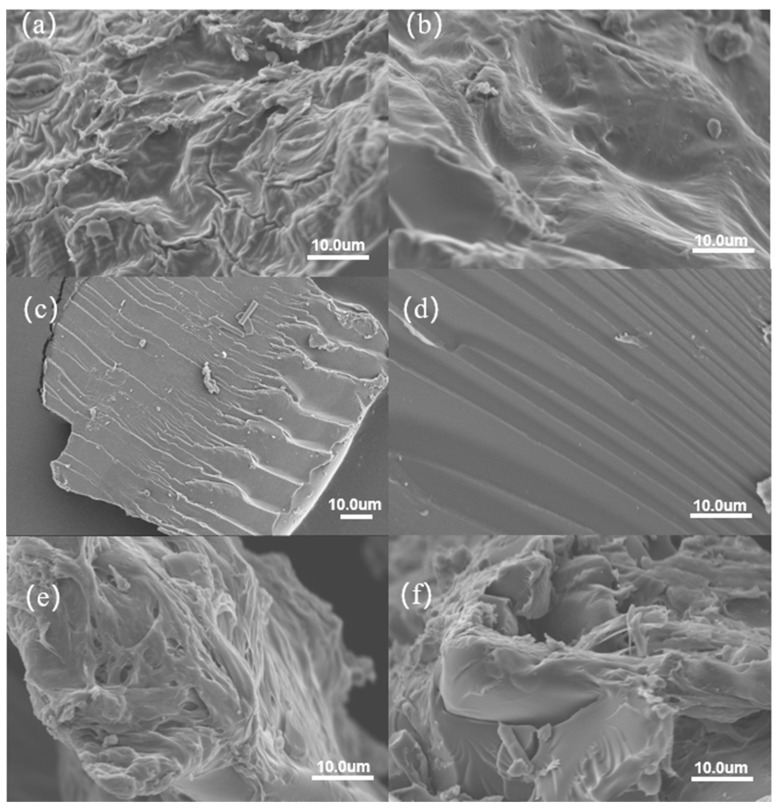
Surface characteristics of (**a**) CW, (**b**) BP, (**c**,**d**) CK, (**e**) CW-(AA-AAm) gel, and (**f**) BP-(AA-AAm) gel.

The surface morphology of CK, BP, CW, BP-(AA-AAm) gel, and CW-(AA-AAm) gel was examined in detail using scanning electron microscopy (SEM). The results demonstrated that the surface characteristics of the CW-(AA-AAm) gel and BP-(AA-AAm) gel were distinctly different from those of the CW, BP, and CK samples, exhibiting markedly dissimilar surface morphologies. As illustrated in Figure 4a,b, the surface microstructure of CW and BP exhibited sparse groove-like stomatal features with a relatively smooth surface texture and a less dense distribution of stomata. Figure 4c,d illustrate the surface characteristics of CK, which exhibit a markedly smooth surface devoid of discernible pore structure or reticulation. As illustrated in Figure 4e,f, the surfaces of both CW-(AA-AAm) gel and BP-(AA-AAm) gel exhibited a multitude of pores of varying dimensions and morphologies, collectively forming a complex and irregular reticular structure. The number of pores and folds in CW-(AA-AAm) gel is significantly larger than that of BP-(AA-AAm) gel. This structural feature endows it with stronger water absorption and water retention properties [24,25].

The results of the SEM analysis demonstrated that CW and BP are not suitable for direct utilization in applications that necessitate effective water management. However, the raw materials were chemically modified to form a complex three-dimensional mesh structure, which not only increased the contact area with water molecules and effectively accelerated the rate of water absorption, but also significantly improved the water retention capacity due to the stability of the mesh structure [26]. The abundance and diversity of pores in the CW-(AA-AAm) gel were particularly noteworthy, with a significantly larger number of pores on its surface than in the BP-(AA-AAm) gel. This structural feature endowed the CW gel with stronger water absorption and water retention properties, demonstrating the significant influence of the raw materials on the water retention properties of the hydrogels.

### 2.4. Analysis of Water Absorption of Hydrogel

**Figure 5:**
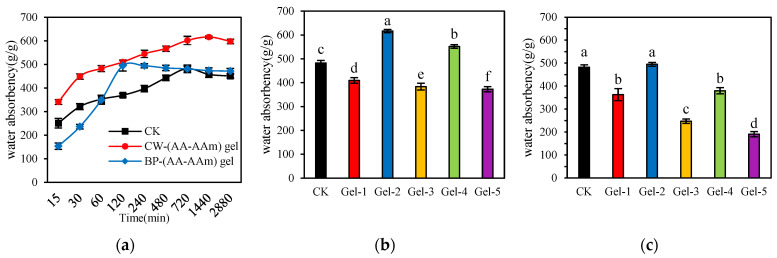
(**a**) Swelling capacity of CK, CW-(AA-AAm) gel, and BP-(AA-AAm) gel at different times; (**b**) Swelling capacity of CW-(AA-AAm) gel under different treatments; (**c**) Swelling capacity of BP-(AA-AAm) gel under different treatments. In the bar graph, letters (a–f) indicate the degree of significant differences among the groups represented by each bar, with “a” denoting the group with the highest level of significance, followed by “b”, “c”, “d”, “e” and “f” representing groups with decreasing levels of significance.

The water absorption of the hydrogels was found to be significantly affected by the various treatments [27]. The addition of MBA at a mass fraction of 0.025% resulted in the highest swelling rate for both CW-(AA-AAm) gel and BP-(AA-AAm) gel in ultrapure water, with values of 616.6 g/g and 495.0 g/g, respectively. In comparison, the swelling rate of CK was relatively low, at 482.4 g/g. The swelling rate of CW-(AA-AAm) gel was markedly higher than that of the other two hydrogels, indicating that it exhibits superior water absorption properties. Figure 5a illustrates that the water absorption rates of CK, CW-(AA-AAm) gel, and BP-(AA-AAm) gel all exhibited a nonlinear increase over time. Initially, they increased rapidly and then gradually stabilized until reaching the saturation absorption rate. The CW-(AA-AAm) gel exhibited the highest swelling rate, reaching 616 g/g after 24 h. In comparison, the BP-(AA-AAm) gel reached its maximum swelling rate in 2 h, while the CK treatment reached 482.4 g/g after 12 h. After reaching the swelling equilibrium, these hydrogels began to slowly release water approximately 12 h later, showcasing their ability to retain and release moisture. As illustrated in Figure 5b,c, the swelling capacity of CW-(AA-AAm) gel was evaluated in comparison with Gel-1, Gel-2, and Gel-3, which exhibited a fixed ratio of AA to AAm, at varying crosslinking densities. It was determined that the equilibrium water absorption ratio (Qeq) of Gel-2 was 616.6 g/g, which was higher than that of Gel-1 (409.6 g/g) and Gel-3 (383.6 g/g). When the ratio of AA to AAm was varied while the cross-linking density was held constant, the Qeq of Gel-4 was 551.8 g/g, which was higher than that of Gel-1 (409.6 g/g) and Gel-5 (373 g/g). These findings suggest that the ratio of monomers plays a pivotal role in influencing the swelling characteristics of hydrogels. In addition, the water absorption of BP-(AA-AAm) gel was markedly inferior to that of CW-(AA-AAm) gel, which may be attributed to the disparities in its molecular structure and crosslinking network [28].

The present study revealed a notable discrepancy in the water absorption characteristics of CW and BP. This phenomenon may be attributed to the cellulose content of both materials and its influence on the water absorption properties. Cellulose, a natural macromolecule, contains a substantial number of hydroxyl groups within its molecular chain. These hydroxyl groups are capable of forming hydrogen bonds with water molecules, which markedly enhances the swelling capacity of the material. The formation of such hydrogen bonds represents a pivotal factor in the water-absorbing capacity of cellulose-based materials. The study conducted by Ribeiro et al. [29] offers compelling evidence that the incorporation of cellulose fibers into silicate polymer composites effectively enhanced the water-absorbing capacity of the materials, thereby underscoring the pivotal role of cellulose fibers in the augmentation of water swelling ability. Additionally, the study by Marcuello et al. [30] indicated that the sensitivity of cellulose-based materials to alterations in humidity may result in modifications to their mechanical behavior in diverse humidity environments. This sensitivity is primarily attributable to the interactions between the hydroxyl groups in cellulose molecules and water molecules, which are intensified with increasing humidity, thereby influencing the water absorption behavior of the material. Furthermore, the findings of Alam et al. [31] indicated that cellulose-based hydrogels exhibit remarkable water absorption capabilities, providing a crucial theoretical foundation for the development of smart cellulose-based hydrogel absorbents. Collectively, these observations highlight the significant potential of cellulose in enhancing the water absorption properties of materials.

The water absorption rates of different hydrogels are closely related to their microstructures and compositions [32]. CW-(AA-AAm) gel exhibits a longer time to reach the peak swelling rate, which is primarily attributed to its complex network structure and higher cellulose and pectin contents. These provide more cross-linking and adsorption sites during water molecule penetration, thereby increasing the swelling ability [33]. However, this also leads to the fact that the water molecules take a longer time to completely penetrate and distribute into the whole structure. The BP-(AA-AAm) gel reached the maximum swelling rate with remarkable rapidity, indicating that its structure is comparatively loose and contains distinctive components that facilitate the rapid absorption of water molecules and the formation of a saturated state [34]. In contrast, the swelling rate of CK is intermediate, and the properties of its base formulation determine the dynamic response of its swelling ability to volume changes.

A comparative analysis of various CW-(AA-AAm) gel treatments revealed that Gel-2 exhibited a markedly higher swelling capacity (Qeq), suggesting that reducing the cross-linking agent dosage can effectively enhance the hydrogel’s water absorption capacity [35]. Furthermore, when the crosslinker ratio was fixed and the AA and AAm were at an equimolar ratio, the hydrogel also exhibited a higher swelling ability. This suggests that the ratio configuration optimized the intermolecular interactions of the hydrogel, which in turn improved the adsorption ability of the water molecules and the stability of the structure [36]. In contrast, the water absorption of BP-(AA-AAm) gel was significantly lower than that of CW-(A-AAm) gel. This discrepancy may be attributed to the bioactive substances (e.g., tannins, flavonoids, etc.) present in banana peels, which exhibit intricate interactions with water molecules and may, to some extent, impede their solubilization properties [37]. This step underscores the significance of raw material selection and cross-linking configuration on hydrogel performance, offering a framework for future material design.

### 2.5. Analysis of Swelling Capacity of Hydrogels at Different pH

**Figure 6:**
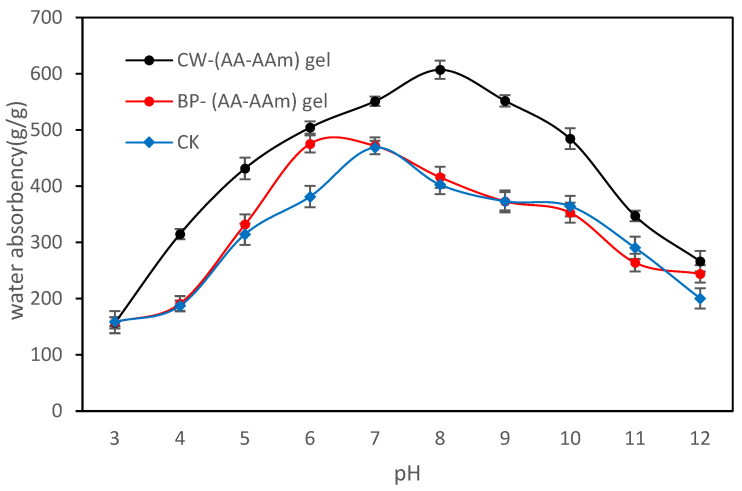
Water absorption curves for CW-(AA-AAm), BP-(AA-AAm), and CK gels at different pH conditions.

In the study, we selected hydrogel samples with the optimal swelling capacity in ultrapure water and evaluated their swelling characteristics in diverse pH solutions (Figure 6). It was demonstrated that CK achieved a maximum swelling rate of 468.8 g/g at a neutral pH (pH = 7), a finding that is closely associated with the intrinsic chemical characteristics and charge distribution of the hydrogel material [38]. CW-(AA-AAm) gel exhibited a swelling rate of 607.7 g/g in a weakly alkaline environment (pH = 8), thereby illustrating its remarkable capacity for water uptake. The highest swelling rate, 475.3 g/g, was observed for BP-(AA-AAm) gel at pH 6. A comprehensive comparison revealed that CW-(AA-AAm) gel exhibited superior swelling ability compared to BP-(AA-AAm) gel and CK across the pH range of 4 to 11, with the measured swelling rates exceeding 300 g/g in all cases. However, the swelling ability of CW-(AA-AAm) gel was found to be significantly enhanced under extremely acidic (pH < 4) and alkaline (pH > 11) conditions. In contrast, the swelling capacity of all three hydrogels was observed to decline markedly in these environments.

Analyses of swelling at varying pH levels demonstrated that CW-(AA-AAm) gel exhibited remarkable swelling performance under weakly alkaline conditions. This was primarily attributed to the incorporation of cellulose, which enhanced the hydrogel’s hydrophilicity [39]. The distinctive molecular structure of cellulose not only facilitates the penetration and retention of water molecules but also optimizes the swelling capacity of the hydrogel under specific pH conditions. The high swelling rate of BP-(AA-AAm) gel at pH = 6, on the other hand, indicates that it maintains its effective water absorption performance under a slightly acidic environment, where substances such as tannins and flavonoids in its composition may be more active and the interactions with water molecules are enhanced [40]. This phenomenon indicates that the incorporation of natural ingredients can effectively enhance the functionality of hydrogels, rendering them more versatile in specific environments. However, the solubility of hydrogels was markedly diminished under both extremely acidic and alkaline conditions. This reduction can be attributed to a shift in ionization equilibrium and the disruption of the network structure [41,42]. In the presence of extreme acidic conditions, the anionic groups present in hydrogels may undergo protonation, which results in a reduction in their overall charge density. This, in turn, leads to a weakening of the interaction between the hydrogel and water molecules. Furthermore, in extreme alkaline environments, the presence of excess hydroxide ions may result in structural damage to the hydrogel and impair its swelling capacity. This finding provides a crucial reference point for the environmental adaptability of hydrogels in practical applications. It underscores the necessity of considering the pH sensitivity of hydrogels when designing and utilizing them, to achieve optimal water retention and stable performance.

### 2.6. Water Retention and Reuse Properties

The preceding experiments demonstrated that among both CW-(AA-AAm) gel and BP-(AA-AAm) gel, the swelling capacity of Gel-2 and Gel-4 exhibited particularly noteworthy performance. To further investigate the water retention characteristics of these two hydrogel types, Gel-2 and Gel-4 were selected for subsequent experimental assessments, with the objective of refining the comparison of their respective differences and advantages in terms of water retention performance.

**Figure 7:**
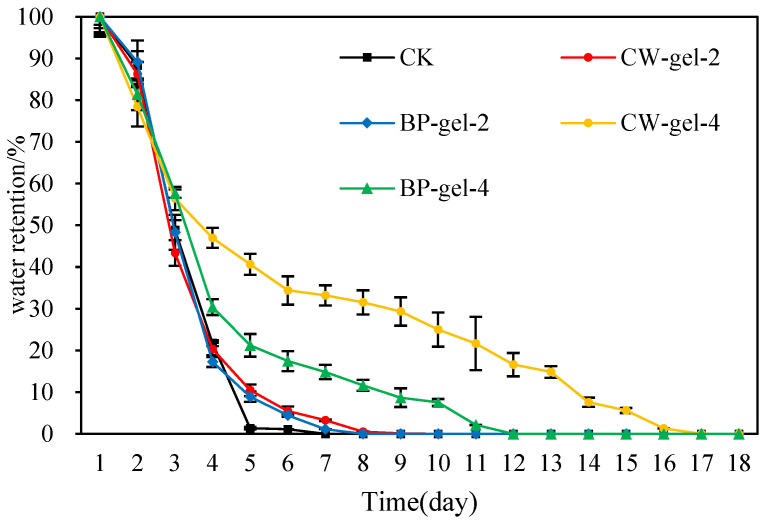
Water retention capacity of various hydrogels under different treatments at room temperature.

As illustrated in Figure 7, CK exhibited the lowest water retention capacity, reaching a constant weight on day 5 only, indicating an almost complete loss of water. In comparison, CW-gel-4 and BP-gel-4 exhibited superior water retention performance, demonstrating the greatest capacity among all tested groups. BP-gel-4 reached a constant weight on day 12, while CW-gel-4 even reached a constant weight on day 17, even after drying at room temperature for up to three days, the water retention was maintained at 57.63%, indicating that they were capable of retaining water for an extended time, which was significantly superior to the other groups. It is noteworthy that despite the higher swelling rate of CW-gel-2 and BP-gel-2, their water retention capacity reached a constant weight on day 8, which was significantly inferior to that of the Gels-4 group. This may be due to the different internal structure of the hydrogel [43]. 

The results of water retention experiments conducted at room temperature indi-cate that CK exhibits the weakest water retention ability, which can be attributed to its inherent structural characteristics. These characteristics result in water retention only for a limited time. In contrast, the excellent performance of CW-gel-4 and BP-gel-4 highlights their advantages in molecular structure and porosity. Although CW-gel-2 and BP-gel-2 have high swelling rates, the lower crosslink density results in a loose in-ternal structure and poor water retention. This conclusion is similar to that of Jahan-dideh et al. [44]. This illustrates the intricate nature of hydrogels in real-world applica-tions, particularly when coupled with the quantity of cross-linking agents, structural integrity, and associated material characteristics [45,46]. It is recommended that fu-ture material designs take these factors into full consideration to enhance the water retention performance of hydrogels in specific applications and provide more stable and effective solutions for agriculture, the environment, and medicine.

**Figure 8:**
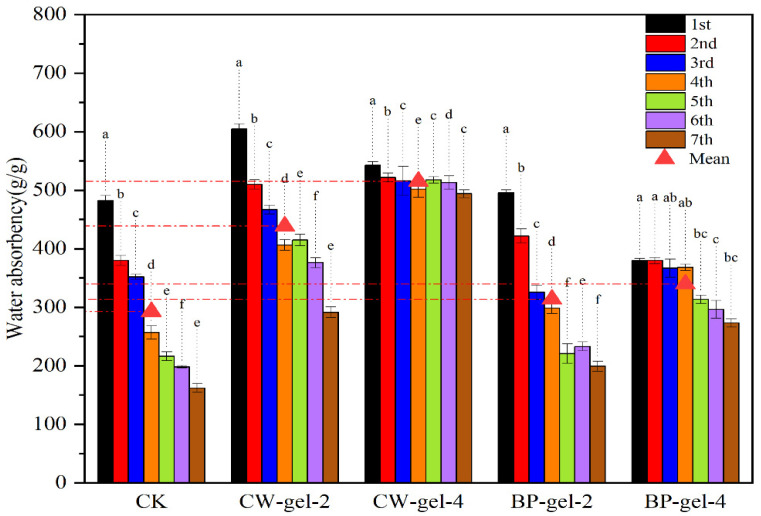
Reuse properties of various hydrogels under different treatments at room temperature. In the bar graph, letters (a–f) indicate the degree of significant differences among the groups represented by each bar, with “a” denoting the group with the highest level of significance, followed by “b”, “c”, “d”, “e” and “f” representing groups with decreasing levels of significance.

Following seven cycles of reuse, the swelling capacity of CK decreased to approximately 33.6%, indicating a notable degradation of the swelling capacity of the base material under repeated cycles of use. In contrast, the swelling ability of CW-gel-4 and BP-gel-4 was found to be as high as 94.6% and 71.8%, respectively (Figure 8). Notably, CW-gel-4 demonstrates remarkable resilience, retaining a near-initial swelling level even after seven cycles of reuse, indicative of its exceptional reuse potential. Furthermore, the swelling capacity of CW-gel-2 and BP-gel-2 was 48.2% and 40.2%, respectively, which was lower overall than that of the Gel-4 group despite exhibiting a certain degree of reuse performance. This suggests that the preparation conditions have a significant influence on the retention of swelling performance [47].

The swelling capacity of CK was found to decrease significantly in the reuse experiments, indicating that the base material exerts a non-negligible influence on the hydrogel performance, particularly in scenarios involving long-term application. In contrast, CW-gel-4 and BP-gel-4 demonstrated enhanced potential for reuse. The superior performance of CW-gel-4 may be attributed to the polysaccharide chain or space conformation formed during its preparation, as well as a higher proportion of stabilizing structural components, which collectively enable CW-gel-4 to maintain its solubilized state more efficiently [48]. Furthermore, the reusability of CW-gel-2 and BP-gel-2 was found to be markedly inferior to that of the Gel-4 group. This indicates that the preparation conditions exert a significant influence on the swelling properties and reusability of hydrogels.

In light of the aforementioned experimental data, CW-gel-4 and BP-gel-4 were identified as exhibiting favorable water absorption and exceptional water retention and reutilization properties. Consequently, these two materials were selected for subsequent soil water retention tests.

### 2.7. Water Retention and Water Holding Capacity of (AA-AAm) Gels in Different Soils

The effects of different types of hydrogels on the water retention and water-holding capacity of various soil types, including sandy, loamy, and clayey soils, exhibited notable differences.

**Figure 9:**
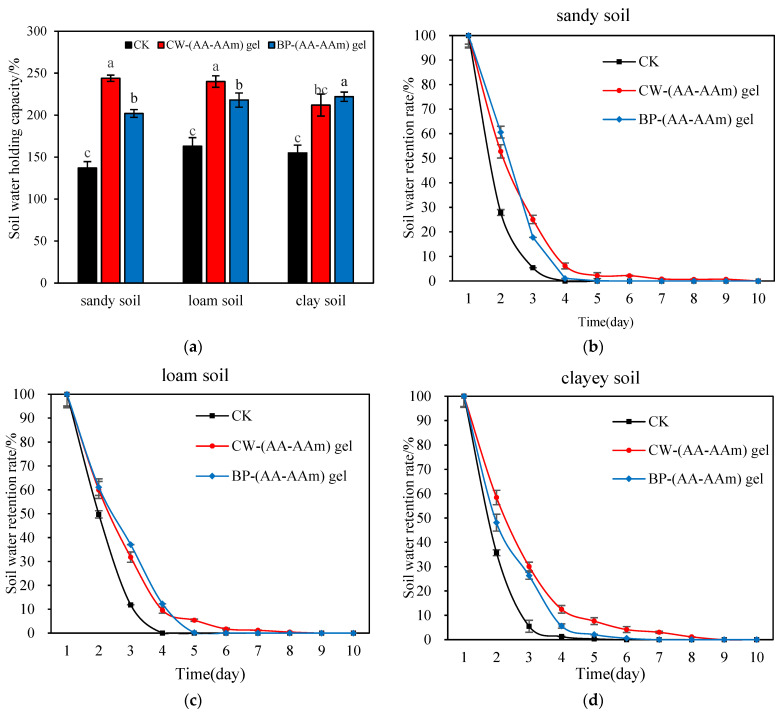
(**a**) Water-holding capacity of CK, CW-(AA-AAm) gel and BP-(AA-AAm) gel added at 0.6% in different soils; (**b**–**d**) Water retention of CK, CW-(AA-AAm) gel and BP-(AA-AAm) gel in different soils. In the bar graph, letters (a–c) indicate the degree of significant differences among the groups represented by each bar, with “a” denoting the group with the highest level of significance, followed by “b” and “c” representing groups with decreasing levels of significance.

As illustrated in Figure 9a, the incorporation of hydrogel markedly augmented the water retention capacity of the soil. Specifically, CW-gel-4 had the most pronounced impact on sandy soils, with a notable increase in water-holding capacity from 137.2% to 244.4%, representing a 78.2% enhancement. The second most significant effect was observed in loamy soils, exhibiting an increase of 47.1%. The smallest effect was observed in clayey soils, which increased from 155.3% to 212.0%, representing a 37.3% increase. In comparison, the effect of BP-gel-4 was more pronounced in clayey soils, with a 43.3% increase in water-holding capacity from 155.0% to 222.6%, and a 6.6% increase in water-holding capacity compared to CW-gel-4. In contrast, the improvement of BP-gel-4 was not significant in sandy and loamy soils, further emphasizing the effectiveness and applicability of different hydrogels in specific soil types.

As illustrated in Figure 9b–d, the incorporation of hydrogel markedly influenced the water retention properties of sandy, loamy, and clayey soils, particularly in sandy soils. In the absence of hydrogel, the water retention period of sandy and loamy soils was observed to be only four days, thereby demonstrating the inherent limitations of their natural water retention capacity [49]. In contrast, the clay soil, due to its unique structural properties, exhibited a water retention period of five days, which is slightly longer than the other two soils, but still has room for improvement. The addition of CW-gel-4 resulted in a significant extension of the water retention period of sandy soils to nine days, representing a five-day increase. Meanwhile, the water retention period of loamy and clay soils was extended to eight days, demonstrating the general effectiveness of CW-gel-4 in improving the water retention performance of the soils, albeit with a relatively modest increase. In comparison, BP-gel-4 extended the water retention period to five days in sandy and loamy soils, and six days in clay soils, which was not as pronounced as the performance of CW-gel-4. However, it demonstrated a distinctive advantage in water adsorption response during the initial stage.

The incorporation of hydrogel has been demonstrated to markedly enhance the soil’s capacity to retain water. Sandy soil is inherently loose and exhibits a diminished capacity to retain water [50]. The incorporation of CW-(AA-AAm) gel, however, enables the formation of a unique structure capable of effectively filling the interstitial spaces between soil particles, thereby enhancing water retention. This effect is most pronounced in sandy soil. In contrast, clayey soil exhibits high water retention capacity, and the addition of CW-(AA-AAm) gel enhanced this capacity, though the effect was not as significant as that observed in sandy soil. The notable improvement in the performance of BP-(AA-AAm) gel in clayey soil can be attributed to its complementary interaction with the structural characteristics of clayey soil, which enhances the retention and transport of water within the soil matrix [51]. This results in the optimal performance of the gel in clayey soil. In contrast, although an enhancement was observed in sandy soils, it was slightly less effective than that observed with CW-(AA-AAm) gel. Therefore, CW-(AA-AAm) gel is more suitable for sandy soils, whereas BP-(AA-AAm) gel is of great value for agricultural and horticultural management in clay soil areas. While the BP-(AA-AAm) gel formulation did not achieve the same degree of water retention as the CW-(AA-AAm) gel, its rapid response to initial moisture levels demonstrated its superiority in enhancing water utilization in the short term. Therefore, BP-(AA-AAm) gel is appropriate for circumstances that necessitate prompt enhancement of soil moisture conditions, whereas CW-(AA-AAm) gel is more suited to long-term irrigation management [52]. This distinction offers direction for future hydrogel selection in agricultural and environmental management, underscoring the significance of material choice in diverse application contexts.

### 2.8. Evaluation of CW-(AA-AAm) Gel for Plant Growth Performance

Given the excellent performance of CW-gel-4 in water retention, it was further employed as a substrate in this study to investigate the effect of the hydrogel at different concentrations (0.0%, 0.5%, 1.0%, 1.5%, 2.0%, and 3.0%) on the growth of wheat seedlings under drought stress conditions.

**Figure 10:**
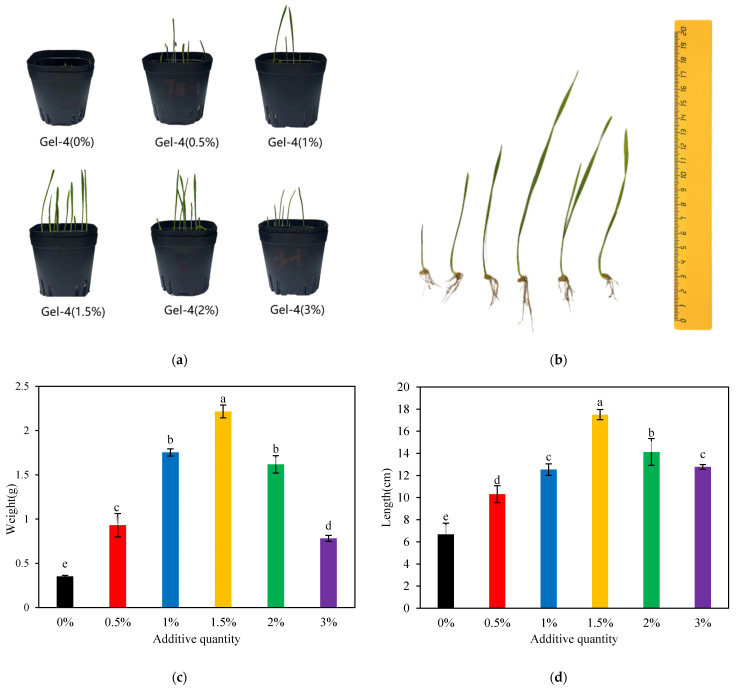
(**a**) Growth of wheat seedlings on day 4 under water restriction; (**b**)Plant height of wheat after cessation of growth at different hydrogel concentrations (0.0% to 3.0%); (**c**,**d**) Mean weight and height for varying hydrogel concentrations. In the bar graph, letters (a–e) indicate the degree of significant differences among the groups represented by each bar, with “a” denoting the group with the highest level of significance, followed by “b”, “c”, “d” and “e” representing groups with decreasing levels of significance.

The experimental results demonstrated that wheat growth was optimal at 1.5% (1.5% CW-Gel-4), followed by 2.0% (2.0% CW-Gel-4), and exhibited a significant improvement compared to the other treatment groups. In contrast, the treatment groups without hydrogel (0.0% CW-Gel-4) and those with a 3% addition (3.0% CW-Gel-4) exhibited the poorest seedling growth (Figure 10). The experimental data presented in this series provides definitive evidence that the addition of an appropriate amount of hydrogel can markedly enhance soil water retention, thereby optimizing the growth environment of wheat seedlings and improving their growth quality and rate.

The application of hydrogels in the context of drought stress has been demonstrated to exert a considerable influence on the growth of crop seedlings. The use of hydrogel in wheat seedling research has demonstrated that an appropriate dosage (e.g., 1.5%) can effectively enhance soil water retention, provide a continuous water supply to the seedlings, and significantly promote their growth. In contrast, the performance of CW-Gel-4 (0.0%) and CW-Gel-4 (3.0%) demonstrated the detrimental effects of inadequate or excessive hydrogel usage [53]. The former resulted in poor seedling growth due to the lack of additional water support, while the latter inhibited normal seedling growth due to the competition for water caused by the excess of hydrogel. This phenomenon underscores the necessity of rationally configuring the quantity of hydrogel incorporated to ensure optimal growth in agricultural applications [54]. The findings of this study provide a significant theoretical foundation and practical reference for agricultural production and water management in arid and semi-arid regions. Further research could investigate the effects of combining different types of hydrogels and crops to optimize water use efficiency and promote sustainable agricultural development.

## 3. Conclusions

In this study, the CW-gel and BP-gel series of composite hydrogels were prepared from CW and BP, respectively. At the lowest crosslink density, the equilibrium swelling rate of the CW-gel reached 616.6 g/g, which was significantly higher than that of the BP-gel, which reached 495.0 g/g. However, both materials exhibited poor reusability and water retention, rendering them suitable only for short-term enhancement of water utilization. When the monomer ratio was held constant, CW-gel exhibited superior reusability and water retention compared to BP-gel, in addition to demonstrating an excellent swelling capacity. This makes it an ideal candidate for use as a soil water retention agent in long-term irrigation management systems. The incorporation of CW-gel and BP-gel into diverse soil types has been demonstrated to enhance the soil’s capacity to retain water, with CW-gel exhibiting the most pronounced effect on sandy soil and BP-gel demonstrating superior performance on clayey soil. The incorporation of hydrogel significantly enhanced wheat seedling growth under drought-stress conditions, with an optimal addition of 1.5%. This finding offers a potential solution for crop growth and water management in arid regions.

As the application of hydrogel technology in agriculture becomes increasingly promising, the topics explored in this study are of significant importance in promoting sustainable agricultural development. In the future, our research team plans to implement a series of in-depth studies, including a detailed analysis of the degradation behavior of hydrogels in soil using burial techniques and environmental monitoring, as well as controlled experiments to assess their potential impact on short-term crop growth [55]. Furthermore, anti-microbial analyses will be conducted to ensure that the negative impact of its degradation products on soil microbial communities is minimized [56]. Based on the findings of our research, we will refine the composition of the hydrogel to improve its biodegradability and compatibility with crops. We are dedicated to collaborating with the agricultural sector and related companies to advance the sustainable application of hydrogels, develop integrated agricultural management strategies, and contribute to agricultural environmental protection and sustainable development.

## 4. Materials and Methods

### 4.1. Materials

The raw materials utilized in this study were CW and BP, procured from a local vegetable market. The chemical reagents employed in this study were acrylic acid (AA), acrylamide (AAm), ammonium persulfate (APS), N, N′-methylene bisacrylamide (MBA), and sodium hydroxide (NaOH), which were manufactured by Tianjin Kemi Chemical Reagent Co. (Tianjin, China) and were of analytical purity.

### 4.2. Preparation of (AA-AAm) Gels

**Figure 11:**
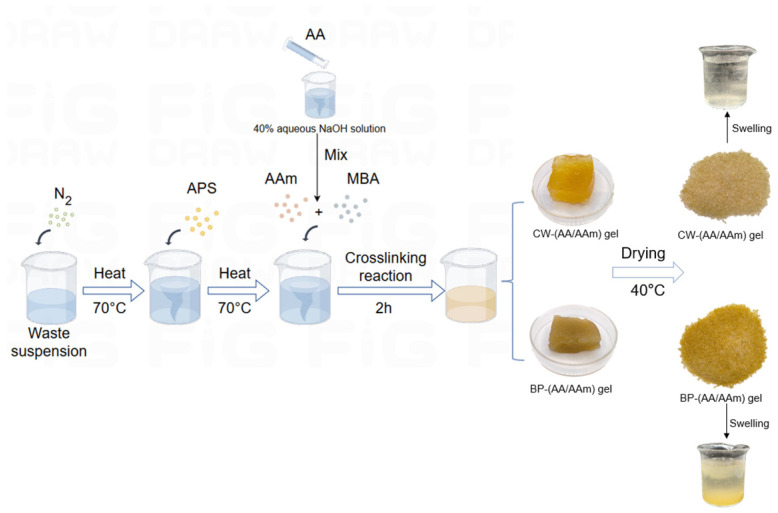
The process of CW-(AA-AAm) gel and BP-(AA-AAm) gel synthesis.

CW and BP were separately added to water, pulverized in a juicer, and then homogenized in a digital disperser to obtain a suspension of the waste. Subsequently, the suspension was introduced to a three-necked flask, which was equipped with a mechanical stirrer and a nitrogen tube. Following a 30-min nitrogen venting period, the suspension was heated to 70 °C, and an amount of initiator APS was added (Figure 11). Following the 30 min, predetermined quantities of AAm, AA (neutralized in a 40% aqueous NaOH solution), and cross-linking agent MBA were introduced [57].

Five grafted polymer hydrogels and one hydrogel devoid of added waste were synthesized from the two materials by varying the proportions of AA, AAm, and MBA, respectively (Table 1). A continuous flow of nitrogen needed to be maintained throughout the reaction. Once the samples had been obtained, they were subjected to a drying process in a dryer set at 40 °C, after which they were ground into powder form for subsequent testing.

### 4.3. Methods of Characterization

#### 4.3.1. FTIR Spectroscopy

FTIR spectra of CW, BP, CW-(AA-AAm) gel, BP-(AA-AAm) gel, and CK were recorded in solid state, and the infrared spectra of the samples were measured using a Fourier Transform Infrared (FTIR) Spectrum Analyzer with rapid pressurization of the powder at a pressure of 10–15 MPa in the wavelength range of 400–4000 cm^−1^ [58].

#### 4.3.2. Morphological Characterization

The microstructure of the samples was investigated by scanning electron microscopy (SEM) (JEOL S-3400N, HITACHI, Tokyo, Japan). In this experiment, the stabilization rate of the electron acceleration voltage was set to 8.0 kV to ensure image quality and reduce errors due to voltage fluctuations. To enhance the contrast of the SEM images, gold plating was employed as a contrast agent.

#### 4.3.3. Swelling Study in Water

Swelling studies of (AA-AAm) gels were carried out using the tea bag method [59]. The ground material was placed in pre-weighed and moistened tea bags, then the hydrogels in the tea bags were immersed in ultrapure water at room temperature for some time to reach the swelling equilibrium, and finally, the tea bags were removed and suspended until no droplets of water fell, and the excess liquid was filtered off with filter paper and weighed. The equilibrium swelling ratio (*Q_eq_*) of the hydrogel is given by the following equation:

(1) $$Q_{eq}=\frac{W_{eq}-W_{0}}{W_{0}}$$

where *W_eq_* is the weight of the swollen sample after achieving equilibrium and *W*_0_ is the weight of the dried sample.

#### 4.3.4. Swelling Study in Different pH

The equilibrium swelling ratios (*Q_eq_*) of (AA-AAm) gels were determined at each pH by placing the gels in solutions with pH values ranging from 2 to 12. Subsequently, the *Q_eq_* of the hydrogels was plotted as a function of pH.

#### 4.3.5. Repeated Swelling Performance

The study entailed determining the stability and solubility of five (AA-AAm) gels in a continuous solubilization–dehydration cycle. Five 0.05 g samples were placed in pre-weighed pouches and the initial mass was carefully recorded. The samples were then immersed in ultrapure water and allowed to stand for 24 h to reach swelling equilibrium and the mass was measured to obtain the swelling ratio m_1_ [60]. The hydrogel samples, which had reached swelling equilibrium, were then transferred to a thermostat, where the temperature was maintained at 80 °C for 6 h. This ensures complete dehydration and assesses the dehydration rate and re-drying potential of the sample. To ensure the accuracy and reproducibility of the data, this comprehensive test protocol was repeated six consecutive times.

#### 4.3.6. Water Holding and Water Retention Studies

In the experiments, the (AA-AAm) gels that had reached swelling study equilibrium were first placed in a room temperature environment (28 °C, 20% humidity), and then the weight of the hydrogels was recorded daily for 20 consecutive days to derive the water retention curve of the hydrogels, and finally, the hydrogels with the best water retention performance were selected for the subsequent experimental measurements.

(AA-AAm) gels with better performance in all aspects were selected to determine their properties in different soils, and the soils used for the experiment were sandy, clay, and loam. Soil samples were collected and combined with 0.6 g of dried hydrogel powder (W_s_) before being introduced to a flowerpot (*W*_0_) containing a tea bag [61]. The experimental materials (flowerpot, tea bag, and mixed samples) were then immersed in ultrapure water for one day. After 24 h, the materials were removed and weighed as W_1_ [62]. Subsequently, it was placed at room temperature and weighed every day to observe the changes defined as *W_t_*, and the dry weight after reaching a constant weight was defined as *W_dry_*, water holding (*W_h_*), and water retention (*W_r_*) curves were plotted from the data.

(2) $$W_{h\%}=\frac{W_{1}-W_{0}}{W_{s}}\times 100$$

(3) $$W_{r\%}=\frac{W_{t}-W_{dry}}{W_{1}-W_{dry}}\times 100$$

#### 4.3.7. Potting Trials

In the study, 30 g of potting soil was weighed and 0.0%, 0.5%, 1.0%, 1.5%, 2.0%, and 3.0% (AA-AAm) gels were added for planting wheat seedlings. Prior to sowing, a series of viability tests were conducted on a randomly selected subset of the seeds. The seeds were subjected to a series of treatments, including removal of surface dirt by water immersion and sterilization. Subsequently, the treated seeds were distributed uniformly within Petri dishes and covered with moist filter paper to maintain optimal humidity. The Petri dishes were then placed in an incubator set to 23 °C and 40% humidity, serving as the germination chamber for the seeds [63]. The results of the experiment demonstrated that the germination rate of the seeds in the batch was 100%. The seeds of wheat seedlings were rinsed three times with water and soaked for 7~10 h. After the seeds were dewy, they were planted into potting soil with hydrogel added and incubated in an incubator at a temperature of 23 °C, and a humidity of 40%; the seeds needed to be sprayed with water every day before germination to ensure that the seeds had sufficient moisture [64]. After the seeds germinated, the water spraying was stopped, and the seedlings were removed from the incubator and placed indoors. The growth of the wheat seedlings was observed and photographs were taken to record it. The height of the plants was measured when the crop stopped growing.

## Figures and Tables

**Table 1:** Feed data for the synthesis of P (AA-AAm) hydrogel raw material.

Number	P (g)	AA (g)	Aam (g)	MBA (g)	APS (g)	NaOH (g)	Water (g)
CK	0.000	40.320	17.280	0.144	1.152	8.960	124.144
Gel-1	28.800	40.320	17.280	0.288	1.152	8.960	124.000
Gel-2	28.800	40.320	17.280	0.144	1.152	8.960	124.144
Gel-3	28.800	40.320	17.280	0.576	1.152	8.960	123.712
Gel-4	28.800	28.800	28.800	0.288	1.152	6.400	126.560
Gel-5	28.800	17.280	40.320	0.288	1.152	3.840	129.120

## Data Availability

Data sharing does not apply to this article as no datasets were generated or analyzed during the current study.
